# The migraine postdrome: Spontaneous and triggered phenotypes

**DOI:** 10.1177/0333102420975401

**Published:** 2021-01-10

**Authors:** Nazia Karsan, Abigail Peréz-Rodríguez, Karthik Nagaraj, Pyari R Bose, Peter J Goadsby

**Affiliations:** 1 Headache Group, Department of Basic and Clinical Neuroscience, Institute of Psychiatry, Psychology and Neuroscience, King’s College London, London, UK; 2 NIHR-Wellcome Trust King’s Clinical Research Facility, SLaM Maudsley Biomedical Research Centre, King’s College Hospital, London, UK; 3 Department of Neurology, Hospital Nuestra Señora Del Rosario, Madrid, Spain; 4 Department of Neurology, Bangalore Medical College and Research Institute, Bangalore, India; 5 Department of Neurology, Auckland City Hospital, Auckland, New Zealand

**Keywords:** Migraine, postdrome, headache, nitroglycerin, trigger, provocation

## Abstract

**Background:**

Non-painful symptoms in migraine following headache resolution can last up to days. Studying the postdrome is important to appreciate the morbidity associated with migraine.

**Methods:**

Fifty-three subjects (*n* = 53) with migraine were studied in an experimental setting, collecting historical phenotypic information on the postdrome in their spontaneous attacks, and also associated with nitroglycerin-triggered attacks, while being observed prospectively. In a separate headache clinic-based cohort of migraineurs (*n* = 42), who were age and sex-matched to the experimental group, the same phenotypic data were extracted from their clinic records. Spontaneous and nitroglycerin-triggered attack phenotypes, and experimental and clinical cohort phenotypes were compared using agreement analysis.

**Results:**

In the experimental group, 100% had a postdrome with their triggered attack, while 98% reported a postdrome in their spontaneous attacks. In the clinical group, 79% had reported a postdrome. In the experimental group, there was good agreement between spontaneous and nitroglycerin-triggered tiredness, hunger, mood change, sensory sensitivities and vertigo and with similarity in premonitory and postdrome phenotypes experienced in the same individual.

**Conclusions:**

The migraine postdrome is common and symptomatically similar to the premonitory phase. The nitroglycerin model and migraine abortive agents can be used to study the postdrome experimentally. Systematic questioning of symptoms, as well as collateral histories from direct observers of migraine attacks, are likely to enhance symptomatic capture of the migraine postdrome, and aid understanding of attack mediation, abortion and neurobiology.

## Introduction

Migraine is more than a disorder of headache; non-painful symptoms associated with the migraine attack, in particular fatigue, have been noted as far back as the 19th century (1). In 1982, it was reported that 47 of 50 migraineurs who used sleep to abort migraine pain still had other symptoms, such as fatigue on awakening (2). A further study showed a heterogeneous cognitive and fatigue postdrome phenotype, with symptoms lasting for a mean duration of 18 h following headache resolution (3). More recent studies have looked at larger study cohorts with various study designs, and generally found that a symptomatically heterogeneous migraine postdrome following headache is common in both adults and children. The symptoms of the postdrome can be divided into four main groups: neuropsychiatric, sensory, gastrointestinal and general (4–8).

For the purpose of this paper, we will use the terminology ‘premonitory’ when referring to the first phase of the migraine attack (9). While the premonitory phase of the migraine attack provides a unique opportunity to study therapeutic agents that may be effective prior to pain onset (10), the postdrome phase allows the study of attack abortion and the effect of acute migraine treatments. In addition, the phenotypic studies have shown the premonitory phase and the postdrome phase of the migraine attack can be symptomatically similar; both are usually dominated by fatigue, mood and cognitive change (7,11). Such similarities have not been formally been studied in migraine, although they suggest these non-painful symptoms may form a continuum pre- and post-headache and are simply less obvious in the presence of severe pain.

The majority of the postdrome studies in the literature have been conducted retrospectively using questionnaires, apart from the Giffin and colleagues study that used prospective reporting through an electronic diary (7). Capturing a spontaneous postdrome reliably has its challenges, including the difficulties patients experience acknowledging the symptoms as being present and being associated with their migraine.

Here we examined nitroglycerin-triggered attacks, which are symptomatically similar to spontaneous attacks (12,13), with good response to usual migraine abortive medication (14). Comparing such data to our clinical patients highlights differences, if any, in prevalence and symptom capture, while accounting for the different types of data collection methods used. Our aim was to document spontaneous and triggered migraine postdrome phenotypes and compare them to each other and to symptoms recorded in a separate cohort of our clinic patients. A secondary aim was to compare spontaneous premonitory and postdrome phenotypes, to assess if there was agreement of similar symptoms. The underlying question being whether the non-painful symptoms associated with migraine form a continuum throughout the attack.

## Methods

Data were collected from subjects who had both a clinical history and symptoms gathered at a nitroglycerin (NTG)-triggered attack (the ‘experimental cohort’) and by review of records for clinical symptoms (the ‘clinical cohort’).

### Recruitment

#### Experimental cohort

Subjects with migraine with and without aura aged 18–50 years were identified through online advertisements, bulletins and patient group advertising through the Migraine Trust, a newspaper advertisement, advertising around the university for staff and student volunteers and through local and national headache clinics. The inclusion criteria for the study included a diagnosis of migraine with or without aura as per ICHD-3 beta, which was in use at the time of the study (15), with up to 22 headache days a month, and no contraindications to study participation and/or nitroglycerin, triptan and non-steroidal anti-inflammatory drug exposure. The headache day limit was selected so the headache frequency was at a level such that study visits could be scheduled on headache-free days to allow triggering of symptoms from a baseline of no pain. We included patients with up to 22 headache days per month, rather than only those with up to 15 days (the currently accepted definition of episodic migraine), to aid recruitment and on the basis that the 15-day limit has no clear biological rationale. Use of any single agent oral preventive therapy for migraine was allowed. Exclusion criteria included medication overuse, use of more than one oral preventive agent for migraine, or the use of neuromodulatory devices, or both, and onabotulinum toxin type A and/or greater occipital nerve injections within the previous 3 months. Illicit drug use and excess alcohol and tobacco consumption were also excluded. Recruitment was completed from February 2015 to July 2017.

#### Clinic cohort

Age and sex-matched migraineurs were selected from patients seen within the Headache Clinics at King’s College Hospital with comparable numbers of baseline headache days and preventive use. Using patient records, information regarding the phenotype of the retrospectively reported postdrome for these patients was collected and tabulated.

### Ethical approval

#### Experimental cohort

The experimental study using nitroglycerin exposure in human subjects was approved by the Camden and King’s Cross Research Ethics Committee in February 2015 (14/LO/2241). All subjects enrolled in the study gave informed written consent for participation, according to the Declaration of Helsinki.

#### Clinic cohort

Data acquired from headache history taking in our clinic were collated as part of a service evaluation.

### Sample size

#### Experimental cohort

We aimed to study 50 subjects to exceed the current highest reported number of subjects exposed to nitroglycerin in the literature (*n* = 44) (16). The study was challenging to conduct, owing to the high screening to eligibility for recruitment ratio that constrained the number.

#### Clinical cohort

There was a challenge in identifying 50 age and sex-matched migraineurs from our clinical cohort meeting inclusion and exclusion criteria for the experimental study, owing to the somewhat skewed diagnoses in patients attending a tertiary headache service. We therefore accepted a smaller sample size (*n* = 42).

### Study conduct

#### Experimental cohort

Three hundred and fifty subjects made email or telephone contact with the study team and were pre-screened for eligibility. Of these 350 subjects, 53 (15%) met eligibility criteria and agreed to attend a screening visit. There was a large pre-screening failure rate, mostly owing to too-frequent headache, preventive medication use and use of other excluded medications.

**Figure 1.**
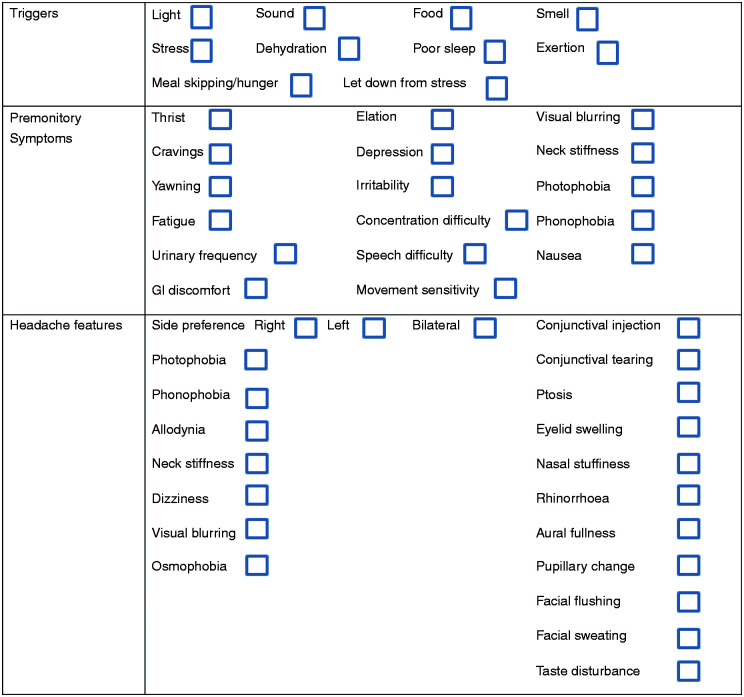
Symptom questionnaire used for phenotypic capture in the experimental study.

All study visits were performed within the Clinical Research Facility at King’s College Hospital. The visit involved written consent for study participation, followed by detailed phenotyping of spontaneous migraine attacks, triggers, medication history and ensuring no medical or pharmaceutical contraindications to any of the study drugs, including nitroglycerin and the acute abortive agents’ intravenous aspirin and subcutaneous sumatriptan. An appropriate cardiovascular and neurological examination was performed. An ECG was performed to exclude cardiac contraindications to nitroglycerin or triptan exposure. Spontaneous migraine attacks were phenotyped retrospectively using the same physician-administered symptom questionnaire used for symptom capture during the triggered attack (Figure 1). Each subject was questioned about symptoms associated with a typical migraine attack for them.

#### Nitroglycerin infusion

Following the history and examination, each subject was exposed to a 0.5 μg/kg/min nitroglycerin infusion over 20 min, to identify those subjects who developed migraine headache. Subjects were symptomatically and haemodynamically assessed with blood pressure, heart rate and oxygen saturation monitoring before the infusion and at 5-min intervals during the infusion, with questioning regarding the evolution of any headache, its site, severity, phenotype and the presence of any other associated migraine symptoms, including premonitory symptoms, using the physician-administered symptom questionnaire shown in Figure 1. Questioning continued at 15-min intervals following the infusion until the time of headache resolution following treatment. All moderate-severe headache meeting criteria for experimental migraine was treated using either 6 mg subcutaneous sumatriptan or 1 g intravenous aspirin, depending on the subjects’ usual abortive response. Triptan responders were treated with sumatriptan, whereas non-steroidal anti-inflammatory responders were treated with aspirin. Injectable formulations were chosen because of their more rapid and potent effect (17,18). In total, information from 53 nitroglycerin-triggered attacks was captured, and premonitory and postdrome symptomatic data from 44, as these subjects successfully had a migraine headache triggered and treated.

#### Attack stage definition

A premonitory symptom was defined as any symptom that the patient reliably experienced before the onset of a migraine headache (excluding typical aura). A postdrome symptom was defined as any symptom that the patient experienced following successful abortion of migraine headache with treatment, in the absence of residual migraine headache, which was typical for a symptom they would experience following a spontaneous migraine headache. Mild head discomfort was allowed.

Subjects had to be completely pain free, and free of any migraine symptomatology (premonitory symptoms, headache, and postdrome symptoms), as well as of any acute migraine abortive medication for at least 12 h prior to a visit. None of the subjects in the study used long-acting triptans or abortive medications that we would expect to continue to have an effect on the threshold of nitroglycerin triggering after 12 h.

#### Clinical cohort

The initial consultation letter from the selected patients’ first encounter at the Headache Clinic documents the headache history in detail. Information on premonitory and postdrome symptoms following either treated or spontaneous attack abortion, as well as baseline headache days, migraine diagnosis and acute and preventive headache therapies were collated retrospectively from clinical notes.

### Statistical analysis

#### Experimental cohort

Statistical analyses were performed using SPSS (version 24). Agreement analysis between spontaneous and nitroglycerin-triggered postdrome symptoms in those who developed migraine following nitroglycerin exposure, and between premonitory and postdrome symptoms in the same individual within the experimental study, was performed using Cohen’s kappa analysis (19).

#### Clinical cohort

For comparison of postdrome phenotypes between the study and clinic cohorts (unpaired data), Chi-square analysis or Fisher’s exact test were used, depending on the counts in each cell of the cross-tabulation.

In all statistical analyses, where relevant, significant results are highlighted with an asterisk (*). When using dichotomous data, the Cohen’s kappa coefficient value is not always reflective of the percentage agreement (20). For this reason, for this study, symptom reporting percentage agreement of 60% or more was considered moderate or acceptable agreement, and these results will also be highlighted when present, irrespective of the Cohen’s kappa value. For the purpose of agreement reporting, > 60% agreement, or kappa > 0.4, or both, were considered significant. The percentage agreement refers to the likelihood of acquiring a yes/yes or no/no response across spontaneous and triggered attacks for each symptom in a 2 × 2 cross-tabulation.

*P* < 0.05 was considered significant.

## Results

### Experimental cohort

We have previously reported the headache phenotypes for this experimental patient cohort (13).

#### Subject demographics

Of the 53 subjects – nine were male, 27 had migraine with aura, 20 had migraine without aura, six had chronic migraine, and 16 (30%) were on single agent preventive therapy. The majority of these (38%) were on a β-blocker, with use of amitriptyline, topiramate, candesartan and pizotifen being less common. The age range of subjects was 18–50 years (mean 36 years), with up to 22 headache days per month (median 8 days, range 1–22 days).

#### Triggering rates

Of 53 subjects, 44 (83%) developed migraine headache following NTG infusion, and each developed a symptomatic postdrome following headache resolution. Fifty-two subjects (98%) developed at least one premonitory symptom following the infusion within 4–155 min following the start (median 23 min).

Headache following the NTG infusion occurred between 20–278 min following the start of the infusion, with a median time of 107 min.

All 44 subjects responded to acute abortive treatment with 6 mg subcutaneous sumatriptan (*n* = 22) or 1 g intravenous aspirin (*n* = 21) within 2 h to pain improvement. Mild residual head discomfort was allowed as a postdrome symptom. The relatively high response rate to abortive treatment in the study is likely due to the injectable formulations and the selection of treatment by prior response to drugs from these classes.

#### Agreement analysis in triggered attacks

The percentage agreement and agreement analysis between spontaneous and triggered attack for postdrome symptoms in the experimental cohort is shown in Table 1. The most commonly reported postdrome symptoms reported spontaneously in patients in the experimental study were neck discomfort (93%), tiredness (75%) and bowel and bladder changes (75%). A median of three postdrome symptoms were reported spontaneously (range 0–6), and four following nitroglycerin-triggered migraine (range 2–5). Those symptoms with the best agreement between spontaneous and the nitroglycerin-triggered attack were tiredness, hunger, mood change and vertigo.

**Table 1.** Agreement analysis between baseline reported postdrome symptoms and following nitroglycyerin triggering in the experimental cohort who developed migraine headache (*n* = 44).

Postdrome symptom	Number reporting symptom at baseline (n)	Number reporting nitroglycerin-triggered symptom (n)	Percentage agreement spontaneous vs. triggered attacks (n/44 and %)		Cohen’s kappa	*P*-value
Neck stiffness	41	22	23/44	52%	0.5	0.6
Tiredness	33	38	31/44*	70%	0.7	0.6
Bowel and bladder changes	33	0	11/44	25%	0	–
Hunger	14	0	30/44*	68%	0	–
Cognitive impairment	10	25	21/44	48%	0.3	0.8
Mood change	8	6	30/44*	68%	0	0.2
Sense of “hangover”	5	20	21/44	48%	0	0.2
Sensory sensitivity (light/sound/smell)	2	17	27/44*	61%	0.3	0.7
Vertigo	4	7	37/44*	84%	0.3	0.05
Head discomfort or movement sensitivity	2	34	10/44	23%	0	0.3

#### Premonitory symptoms compared to postdrome symptoms in triggered attacks

There were more premonitory symptoms compared to postdrome symptoms reported spontaneously without nitroglycerin (mean difference 1.3 symptoms, *t_52_* = 4.3, *P* < 0.001), and similarly following nitroglycerin (mean difference 1.3 symptoms, *t_43_* = 3.3, *P* = 0.002). There was a weak positive correlation between the number of headache days at baseline and the number of spontaneous postdrome symptoms (Pearson correlation 0.3, *P* = 0.04), although not for premonitory symptoms (Pearson correlation 0.18, *P* = 0.19).

#### Premonitory symptoms compared to postdrome symptoms in spontaneous attacks

The premonitory and postdrome symptom comparison data is shown in Table 2. For many symptoms, there was acceptable (kappa > 0.4 or percentage agreement > 60%) agreement between symptom reporting during the premonitory and postdrome phases of spontaneous migraine attacks.

**Table 2.** Agreement analysis between premonitory and postdrome symptoms reported at baseline in the experimental cohort (*n* = 53) at baseline, in the absence of nitroglycerin.

Symptom	Number reporting premonitory symptom (n)	Number reporting postdrome symptom (n)	Percentage agreement premonitory and postdrome symptom (%)	Cohen’s kappa	*P*-value
Tiredness	45	52	46/53 87%*	0.3	0.01
Nausea	10	6	45/53 85%*	0.4	0.001
Photophobia	15	6	42/53 79%*	0.4	0.001
Cognitive impairment	38	48	36/53 85%*	0	0.7
Neck stiffness	32	4	27/53 51%	0.05	0.4
Hunger	16	4	37/53 70%*	0	0.2
Mood change	35	17	29/53 55%	0	0.5

### Clinical cohort

#### Subject demographics

Of the 42 subjects, seven were male. Fourteen (33%) had episodic migraine without aura, 15 had episodic migraine with aura (36%) and 13 had chronic migraine (31%). Twenty-three (55%) were on single agent preventive therapy; the most common being candesartan (52%), with use of flunarizine, tricyclic antidepressants, propranolol, topiramate and gabapentin being less common. The age range of subjects was 18–50 years (mean 35 years), with up to 21 headache days per month (median 9 days, range 1–21 days).

#### Symptom phenotype

Thirty-three patients reported at least one postdrome symptom associated with their attacks (79%). There was a range of between 0–4 postdrome symptoms reported (median 1 symptom). The most common symptoms were tiredness (64%), cognitive difficulty (33%) and neck stiffness (17%). Forty patients reported at least one premonitory symptom (95%). There was a range of between 0–7 premonitory symptoms reported (median three symptoms). The most common symptoms were concentration trouble (69%), fatigue (50%) and mood change (45%).

#### Agreement analysis

Within the clinical cohort, there was no agreement between reporting of the same premonitory and postdrome symptom in the same individual for any symptom tested. There was a weak negative correlation between the number of headache days at baseline and number of premonitory symptoms reported (Pearson correlation −0.3, *P* = 0.05) and similarly for postdrome symptoms (Pearson correlation −0.3, *P* = 0.02). There was no significant correlation between the number of premonitory and postdrome symptoms reported (Pearson correlation 0.3, *P* = 0.08).

### Comparing the experimental and clinical cohorts

When the postdrome and premonitory symptom phenotypes were compared between the clinical cohort and the experimental cohort’s spontaneous attacks, there was no significant association between the symptoms reported in both cohorts, apart from for postdrome vertigo and sensory sensitivities, with generally richer premonitory and postdrome phenotypes within the experimental cohort compared to the clinical cohort (Table 3).

**Table 3:** Agreement analysis between premonitory and postdrome symptom reporting at baseline (spontaneous attacks) in both the experimental and clinical cohorts.

Postdrome symptom	Number reporting spontaneous symptom during study (n/53, %)		Number reporting symptom from clinic cohort (n*/*42, %)		*p*-value (Chi-square/Fisher’s exact)
Neck stiffness	48/53	91%	7/42	17%	1
Tiredness	38/53	72%	27/42	64%	0.3
Bowel and bladder changes	37/53	70%	0/42	0%	1
Hunger	15/53	28%	1/42	2%	0.6
Cognitive impairment	11/53	21%	14/42	33%	1
Mood change	8/53	15%	3/42	7%	1
Sense of hangover	6/53	11%	1/42	2%	1
Sensory sensitivity (light/sound/smell)	4/53	8%	2/42	5%	<0.001*
Vertigo	4/53	8%	1/42	2%	0.04*
Head discomfort or movement sensitivity	3/53	6%	4/42	10%	1

Premonitory symptom	Number reporting spontaneous symptom during study (n/53, %)		Number reporting symptom from clinic cohort (n*/*42, %)		*p*-value

Mood change	30/53	57%	21/42	50%	0.7
Fatigue	42/53	79%	21/42	50%	1
Cognitive impairment	45/53	85%	25/42	60%	0.7
Yawning	28/53	53%	7/42	17%	0.4
Photophobia	15/53	28%	1/42	2%	1
Neck stiffness	30/53	57%	16/42	38%	0.9
Food cravings	15/53	28%	4/42	10%	1
Thirst	16/53	30%	4/42	10%	0.6
Nausea	10/53	30%	2/42	5%	1

## Discussion

Here, we report the detailed phenotype of postdrome symptoms in an experimental study cohort reported at baseline and following nitroglycerin provocation, and further compare this to a clinic-based cohort. Despite different methods of retrospective and prospective data collection, the migraine postdrome is common, nitroglycerin-triggering rates of migraine with a postdrome are high and the response to migraine abortives is good. The data suggest the nitroglycerin model a suitable experimental means of studying the migraine postdrome. The agreement between spontaneous and triggered symptomatology was acceptable for some symptoms; however, it was poorer than compared to previously reported premonitory and headache phenotypes with the same model (13). The likelihood of reporting a spontaneous postdrome symptom associated with a typical migraine attack and having the same symptom triggered with nitroglycerin was good, although less so than for premonitory or headache symptoms.

The migraine postdrome is considerably less studied than the premonitory phase, the prevalence is variable and phenotype heterogeneous, with up to 255 symptoms described by Blau in the earliest reports (2,3,21), and several others over the years also commenting on this resolution phase (5,22–26). Patients may fail to recognise non-painful symptoms following headache resolution, deem them to be expected following pain, and attribute them to the treatment taken for headache abortion. For these reasons, it is possible that the differences between the spontaneous and triggered phenotypes are due to the differences in symptom capture within the experimental study, with retrospective recall of spontaneous attacks and prospective reporting and physician observation of triggered attacks. Unfortunately, prospective study of postdrome symptoms with spontaneous attacks requires use of patient-recorded diary data, which was outside the scope of this study. If subjects had failed to recognise some symptoms as being associated with spontaneous attacks, there may have been additional, or indeed alternative symptoms identified during the experimental study when specifically questioned about particular symptoms. Environmental factors and differences between the experimental study and spontaneous attacks in patients’ own surroundings are also likely to have contributed to these differences. Whilst the spontaneous phenotype in the experimental study was captured using the symptom questionnaire, the phenotype amongst the clinical patients was collected from offering of symptoms by patients, or specific questioning by the physician taking the history. Clinic history taking was not as standardised over the time that the clinic patients were identified, may have varied between headache physicians and may well have changed over time, given the increased standardisation of systematic questioning in our headache history-taking as our interests and potential research questions have evolved with time (24).

When the phenotype of the spontaneous premonitory phase and postdrome were compared in the same individual within the experimental study, there was acceptable agreement for the majority of symptoms. This was not the case when compared to any symptoms reported in the clinical cohort. Again, we feel that this is likely a reporting issue within our clinic as we were largely relying on patient retrospective recall and open questions (“how do you feel when your migraine headache has settled?”). Our questioning has evolved over time, and now largely constitutes a similar questionnaire to that used in the experimental study (“do you experience fatigue, cognitive change, neck discomfort etc following headache resolution and how long do these symptoms last for?”). However, when systematically questioned within the experimental study, the phenotypes of the premonitory phase and the postdrome within the same individual during spontaneous attacks do seem similar. The result suggests these symptoms form a continuum, rather than occurring in specific defined phases, perhaps being less noticeable during headache. Whilst functional imaging, electrophysiology, visual processing and sensory responses have identified distinct biology during the premonitory phase (27), systematic information on the postdrome is not available. Further studies will no doubt inform the understanding of attack termination and the neurobiological basis of the postdrome.

The results suggest those with more baseline headache days would be more likely to experience more postdrome symptoms. This is not entirely unexpected, since those with more headache burden may have a more enriched postdrome phenotype owing to baseline disease activity. Unfortunately, phenotype and functional brain imaging studies in this phase are sparse, and to our knowledge, the biology of migraine amongst individuals with different numbers of headache days a month has not been studied before beyond the episodic and chronic criteria. For the experimental cohort, subjects were only permitted to attend the study visit if they were completely free of any migraine-related symptoms and had been for at least 12 h, to prevent capture of a spontaneous premonitory phase or postdrome on a study visit. In this setting, all postdromes were directly observed by the physician and followed abortive therapy. Each subject was asked to contact us the evening of the study visit after leaving the facility or the next day if they had rebound headache, or arguably another migraine headache following a possible symptomatic premonitory phase prior to another attack; this did not pose an issue. Unfortunately, in the clinical cohort, this was more difficult to dissect, although, in general, patients reported distinct symptoms following a headache and others warning them of a headache. This is clearly an area that warrants further investigation, particularly in the higher frequency episodic and chronic migraine patients. The premonitory phenotype reported was considerably more heterogeneous than the postdrome across both the experimental and clinical cohorts, contrary to the available literature. Despite the differences, the dominance of arousal, mood, cognitive and homeostatic mechanisms in both is interesting, and further alludes to a similar neurobiological basis to both phases.

### Limitations

The data collection between spontaneous and triggered attacks in the experimental cohort was not the same and this must be considered, although it is inherent in each dataset. Similarly, data collection from clinic histories have significant limitations, which must temper conclusions. Moreover, environmental differences may account for some variation in reporting, as, for example, cognitive change may well be more noticeable in one’s own surroundings compared to during a study visit, while sensory sensitivities may be more noticeable during a study visit compared to in one’s normal surroundings. We did not use the exact abortive treatment that the subject would usually use to terminate headache; we did try to administer an agent in the same class. Clearly, the differences in route of administration, dose and in some cases differences in agent used, could have had an impact on the phenotype of symptoms experienced following treatment. Drug treatment itself is an important confounder to appreciate but studying treatment-naïve spontaneous postdromes experimentally with standardisation of post-headache behaviours is both ethically and logistically challenging. We did not specifically account for potential drug side effects when capturing the postdrome, although immediate effects such as flushing after sumatriptan administration, and epigastric discomfort after aspirin administration, were excluded as true postdrome symptoms, and only symptoms occurring at a delay and following headache improvement were documented.

The recruited subject cohorts were heterogeneous and included those with and without aura and those on preventive therapy. We did not have sufficient statistical power in each group to compare the postdrome phenotypes, and it is possible that baseline preventive use had an effect on symptoms experienced. We used two abortive treatments to treat the headache following nitroglycerin and did not have sufficient sample sizes to compare formally the postdrome phenotypes following each treatment, with a similar issue within the clinical group with a lack of standardisation of abortive treatment used. In the experimental cohort, due to the nature of such treatments, not every subject was suitable for the same treatment, and we were not keen to expose subjects to a new treatment for them that did not have proven efficacy for their attacks. Going forward, studying more distinct and defined patient groups, and studying postdrome phenotypes following specific migraine abortive treatments is planned.

Within the clinical study, we identified limitations to our historical data collection regarding the non-painful symptoms associated with migraine (24) and had begun to consider them. Certainly, the clinic cohort data collection was retrospective, as any spontaneous postdrome must be. The sample size for the clinical cohort was smaller, owing to the nature of a tertiary headache service and issues identifying age and sex-matched patients to experimental studies. In the future, we plan to evaluate systematically our clinic patients again after a period of using a questionnaire, to see if we observe a more detailed premonitory and postdrome phenotype. It is clear that systematic questioning as was performed in the study, as well as prospective observation of attacks, can lead to identification of an enhanced migraine phenotype. Therefore, similar questioning during history taking, as well as collateral histories from family and friends about the behaviour of a migraineur before, during and after headache, should be considered. The drug treatments used by patients in the clinical study were not always the same across different attacks and many patients had not tried the experimental study treatments before, having used oral options. In addition, it is not clear whether each patient reported a spontaneous or treated postdrome, as this question was not specifically asked in the history. We would argue that outside of drug effects and possibly the duration of the postdrome, the postdrome phenotype following headache resolution with headache abortive treatment or with spontaneous or sleep-induced headache resolution should be similar, although this poses further interesting questions about how and why a migraine attack stops, an area that warrants further work going forward.

## Conclusion

This study provides evidence to support the role of the nitroglycerin model in studying the migraine postdrome, if treatment effects are taken into account. In addition, there is some suggestion that premonitory and postdrome phenotypes are similar in the same individual. Non-painful symptoms in migraine may, therefore, be mediated through a continuum of brain activity changes in areas controlling arousal, cognition, and homeostasis. It is clear that systematic and standardised questioning of patients, though posing a potential source of reporting bias, can lead to enhanced and more detailed migraine phenotypes, as can direct observation of migraine attacks. This is important for physicians to recognise and consider for history taking going forwards, to appreciate the prevalence and phenotype of the postdrome, the effects, if any, of different migraine abortive medications on its manifestations and the depth and duration of disability caused for the patient. Further understanding of this important part of the migraine attack is vital to appreciating the neurobiological mechanisms of pain abortion, brain “recovery” to its interictal state, and therapeutic mechanisms for pain.

## Article highlights

- The nitroglycerin model is an effective means of experimentally studying the migraine postdrome, if confounding treatment effects are taken into account.
- The agreement in postdrome phenotype between spontaneous and triggered attacks is less impressive than for other migraine symptoms, perhaps owing to the comparatively poor recognition of postdrome symptoms and their phenotype by patients, differences in phenotypes dependent on drug agent used to treat pain, or spontaneous pain abortion, and reporting bias within the study accompanied by prospective observation of attacks.
- The phenotype of the postdrome seems less heterogeneous than the premonitory phase, but there is a similarity in brain systems involved in both, including arousal, cognition and homeostasis.
- There is a suggestion that, in the same individual, the phenotype of premonitory and postdrome symptoms is similar, alluding to the possibility that premonitory symptoms begin prior to headache and perhaps persist throughout pain and following headache resolution in a symptom continuum.
- There are differences in migraine phenotypes between experimental and clinical patient cohorts, in some part owing to differences in symptom capture and reporting, and these suggest that systematic and detailed questioning regarding migraine symptoms in the clinic is important in truly appreciating the breadth of the migraine postdrome, its associated disability, and the effects of migraine abortive agents.
